# Pollination biology of *Impatiens capensis* Meerb. in non-native range

**DOI:** 10.1371/journal.pone.0302283

**Published:** 2024-06-20

**Authors:** Agnieszka Rewicz, René Monzalvo, Monika Myśliwy, Grzegorz Tończyk, Andrea Desiderato, Saroj Ruchisansakun, Tomasz Rewicz

**Affiliations:** 1 Department of Geobotany and Plant Ecology, University of Lodz, Łódź, Poland; 2 Molecular Systematics Laboratory, Autonomous University of Hidalgo State, Biological Research Center., Carboneras, Mineral de la Reforma, Hidalgo, México; 3 Institute of Marine and Environmental Sciences, University of Szczecin, Szczecin, Poland; 4 Department of Invertebrate Zoology and Hydrobiology, University of Lodz, Łódź, Poland; 5 Department of Plant Science, Faculty of Science, Mahidol University, Bangkok, Thailand; Universidade Federal de Uberlandia - Campus Umuarama, BRAZIL

## Abstract

Pollination biology in the widespread species *Impatiens capensis* Meerb. has only been studied in America, specifically in zones of the U.S.A. and Canada. In this study, we investigated the pollination biology of *I*. *capensis* using an integrative identification approach using morphological and molecular tools in four populations of Northwest Poland. We also determined and compared the functional characteristics of the pollinators of the introduced species from the study sites and the native ones reported, for the latter collecting information from bibliographic sources. Visitors were identified using standard morphological keys, including identifying and classifying insect mouthparts. Molecular identification was carried out using mitochondrial DNA’s cytochrome oxidase subunit I (COI). We morphologically identified 20 species of visitors constituted by 17 pollinators and three nectar robbers. DNA barcoding of 59 individuals proved the identification of 18 species (also 18 BINs). The frequency of pollinator species was primarily made up of representatives of both Hymenoptera (75%) and Diptera (21%). The morphological traits, such as the chewing and sucking mouthparts, small and big body height, and robber and pollinator behavior explained mainly the native and introduced visitors’ arrangements that allow pollination success. However, to understand the process comprehensively, further investigation of other causalities in pollination success and understanding the diversity of pollinators in outer native ranges are necessary.

## Introduction

*Impatiens* Linnaeus is the largest genus in the Balsaminaceae family, containing approximately 1000 species with new species discovered constantly [1–3]. It is known for its high variability of traits [4–8], but also widespread distributions [9], with annual or perennial cycles of growing [10, 11]. Recent systematic and phylogenetic studies of the genus placed *Impatiens* as a sister group of the monotypic genus *Hydrocera* Blume ex Wight & Arnott [8]. *Impatiens* is actually divided into two subgenera: *Impatiens* (including seven sections: *Uniflorae*, *Scorpioidae*, *Tuberosae*, *Impatiens*, *Fasciculatae*, *Racemosae*, *Semeiocardium*), and *Clavicarpa* [8]. The genus *Impatiens* is distributed primarily in the Old World tropics and subtropics in five hotspots: Africa (c. 131 spp.), Madagascar (c. 260 spp.), Southern India with Sri-Lanka (c. 200 spp.), Sino Himalaya (c. 140 spp.) and Southeast Asia (c. 300 spp.) [5, 12–14]. These hotspots exhibit heightened levels of local endemism. For instance, up to 91% of species in southern India are endemic, as reported by Rao et al. [15]. Studies conducted in the temperate zones of Europe [9] North America [16–18] indicate a lower species richness.

*Impatiens capensis* Meerb. (= *I*. *biflora* Walter, *I*. *fulva* Nutt), described by Merburg in 1775 in his “*Afbeeldingen van zeldzaame gewassen*”, is known as the orange jewelweed, jewelweed, orange balsam, or spotted touch-me-not, and is one of the five North American native *Impatiens* species. Its native distribution in the Eastern part of North America spans from Florida in the U.S.A. to Quebec in Canada [19, 20]. Although it was introduced to the west coast of North America [21], and recently has been reported to expand towards the lowlands of Northwestern Oregon, Western Washington, and Southwestern British Columbia [22]. Due to its capability to adapt to different conditions [23, 24], this species was able to successfully establish permanent and vital populations in several new countries outside the native range (e.g., Japan and México [25–27]). However, most of the new records and documented expansions are known from Europe, and occurring nowadays in eight countries (i.e., U.K., the Netherlands, France, Belgium, Finland, Denmark, Poland, and Germany) [28, 29].

Invasive plants can cause irreversible changes in the structure, functioning, and services of ecosystems, often reducing the distribution or even leading to the extinction of native species [30]. These effects result from the intense competitive impact of invasive plants, which can gain an advantage over native flora, introducing new ecosystem dynamics [31]. Also, invasive plants can be vectors for pathogens, further increasing pressure on existing ecosystems, and resulting in potentially irreversible consequences for local biodiversity [32]. Understanding the mechanisms of plant invasions and developing effective management strategies are becoming crucial to protect ecosystems against the adverse effects of the introduction of non-native plant species [33–35]. In this study, definitions according to [36] have been adopted, categorizing species as naturalized i.e., indicating non-native plants that consistently reproduce and maintain populations over multiple life cycles without direct human intervention, or despite such intervention. These plants typically recruit offspring in proximity to adult plants and may not necessarily spread to new natural or seminatural ecosystems. Conversely, the term invasive is applied to those naturalized plants that generate reproductive offspring, often in significant quantities and at considerable distances from parent plants. This dispersal potential, with approximate scales exceeding 100 meters for taxa spreading by seeds over less than 50 years, or over six meters every three years for taxa spreading by roots or creeping stems, highlights their capacity to spread across substantial areas [36]. As its propagation outside the native range has shown negative effects on other native congeners [37], the interactions with native flora must be appropriately addressed.

Pollination biology in *Impatiens* spp. has been discussed by several studies, from pollination systems to reproductive syndromes and the evolution of flower traits. Recently, Ruchisansakun et al. [38], considering works under a pollination-systems perspective, proposed a reconstruction of the evolution of pollination syndromes and divergent use of the same pollinator in *Impatiens* based on 58 species. *Impatiens* pollination biology comprehends investigations in the four continents of the known distribution of the genus, including native and non-indigenous species (NIS—referred to human unintentional and deliberate species introduction outside of their natural past and present range, as well as in their natural dispersal potential [39]). Particular attention was paid to native species from Asia [40–44], Africa [5, 45–47], America [48–50] and Europe with fewer investigations [9]. Nevertheless, studies on species such as *I*. *glandulifera*, *I*. *balflourii*, and *I*. *parviflora* showed contrasting results regarding the interaction with surrounding flora and fauna (i.e., positive, neutral, or negative interaction) [51–55]. *Impatiens* species may present two reproduction mechanisms: cleistogamy (i.e., self-pollination), in which seeds production occurs in organisms with perianth closed, and often possessing structures with size reduction [56, 57], and chasmogamy (i.e., cross-pollination), through abiotic (e.g., wind) and biotic (e.g., animals) vectors. For the latter, also called zoogamy, several species of pollinators of *Impatiens* spp. are known, such as birds (e.g., sunbirds and hummingbirds) or different insects (e.g., butterflies, bees, and flies), being both generalist or specialist [5, 49, 58, 59].

The pollination process in chasmogamic individuals of *I*. *capensis* includes effective pollinators and robbers, mostly invertebrate organisms, like bees, flies, butterflies, beetles, mosquitoes, and ants, while for vertebrates, the hummingbirds represent the unique visitors. Furthermore, visitors with other interactions (plant consumers, parasites, pollinator predators inhabiting the plant) have been reported, both invertebrates (e.g., aphids, beetles, grasshoppers, slugs, and spiders) and vertebrates (e.g., deers, gamebirds, mice, and shrews). Visitor studies in *I*. *capensis* have been carried out only in America, principally on populations from the U.S.A. and limited studies in Canada (see S1 Table—*I*. *capensis* visitors (with literature sources) in supplementary information).

DNA barcoding [60] is a molecular technique used to identify and classify species by analyzing a standardized fragment of the mitochondrial cytochrome oxidase I (COI) gene, widely applicable across both invertebrates and vertebrates [61, 62]. It provides a rapid and reliable method for species identification, crucial in biodiversity studies, ecology, conservation, and forensic investigations. This technique involves comparing sequences with a reference library, ensuring accurate species identification even for morphologically similar or cryptic species [63–65]. The molecular identification of pollinators through DNA barcoding is particularly significant in pollination biology studies [66]. It offers precise and swift species identification, especially valuable when studying diverse pollinator communities [67, 68]. This knowledge contributes to understanding the dynamics of pollination networks, illuminating the mutualistic relationships between pollinators and plants, and identifying key species critical for ecosystem functioning [69–71].

The current study aims to 1) identify visitors outside the native range of *I*. *capensis* using morphological and molecular tools, 2) quantify true pollinators and robbers, and 3) compare the functional traits of pollinators from native and invaded ranges. We hypothesized that most visitors would exhibit complementary traits to the flower morphology, such as bees, bumblebees, and wasps (bees pollination syndrome), indicating functional similarities between pollinator communities.

## Materials and methods

### Morphological description of *I*. *capensis* flower

*Impatiens capensis* plants produce chasmogamous and cleistogamous (self-fertilizing, non-opening) flowers. Cleistogamous flowers are green, with a reduced size of anthers and sepals; with lack nectaries and the petals [56]. The chasmogamous flowers are deep orange to yellowish-orange and have orange to reddish-orange spots [22]. The flowers measure 20 to 25 mm in length and consist of three sepals and five petals. Among the three sepals, one is enlarged, saccate, and shapes a recurved spur, while the remaining two are notably smaller. The saccate sepal exhibits an abrupt and convex taper leading to the spur and contains nectar [18]. One petal creates the upper lip of the flower, and two fused petals make up the lower lip, which is divided into two lobes and serves as a landing pad for visiting insects. Moreover, two smaller lateral petals are situated between the upper and lower lips of the flower [72]. The stamens are fused above the pistil, and after the pollen is released, they fall, exposing the stigma of the pistil [56].

### Study areas

Since the confirmation of its presence in Poland in the 1980s, *I*. *capensis* has been spreading throughout the Western Pomerania region, particularly around the Szczecin Lagoon, Lake Dąbie, and the lower section of the Oder River, as well as neighboring areas, infiltrating moist forests, thickets, riparian herbaceous communities, and ruderal environments along ditches and watercourses [19, 73, 74]. The research area is influenced by a dynamic interaction of oceanic and continental climates. The average annual air temperature is 9.5°C; the summer temperature averages 18°C, while in winter, it is 1°C. The proximity of the Baltic Sea strongly influences the relative humidity of the air, which averages 78–80%. The average annual precipitation is 600 mm, and the average duration of the growing season is 235 days [75].

**Fig 1:**
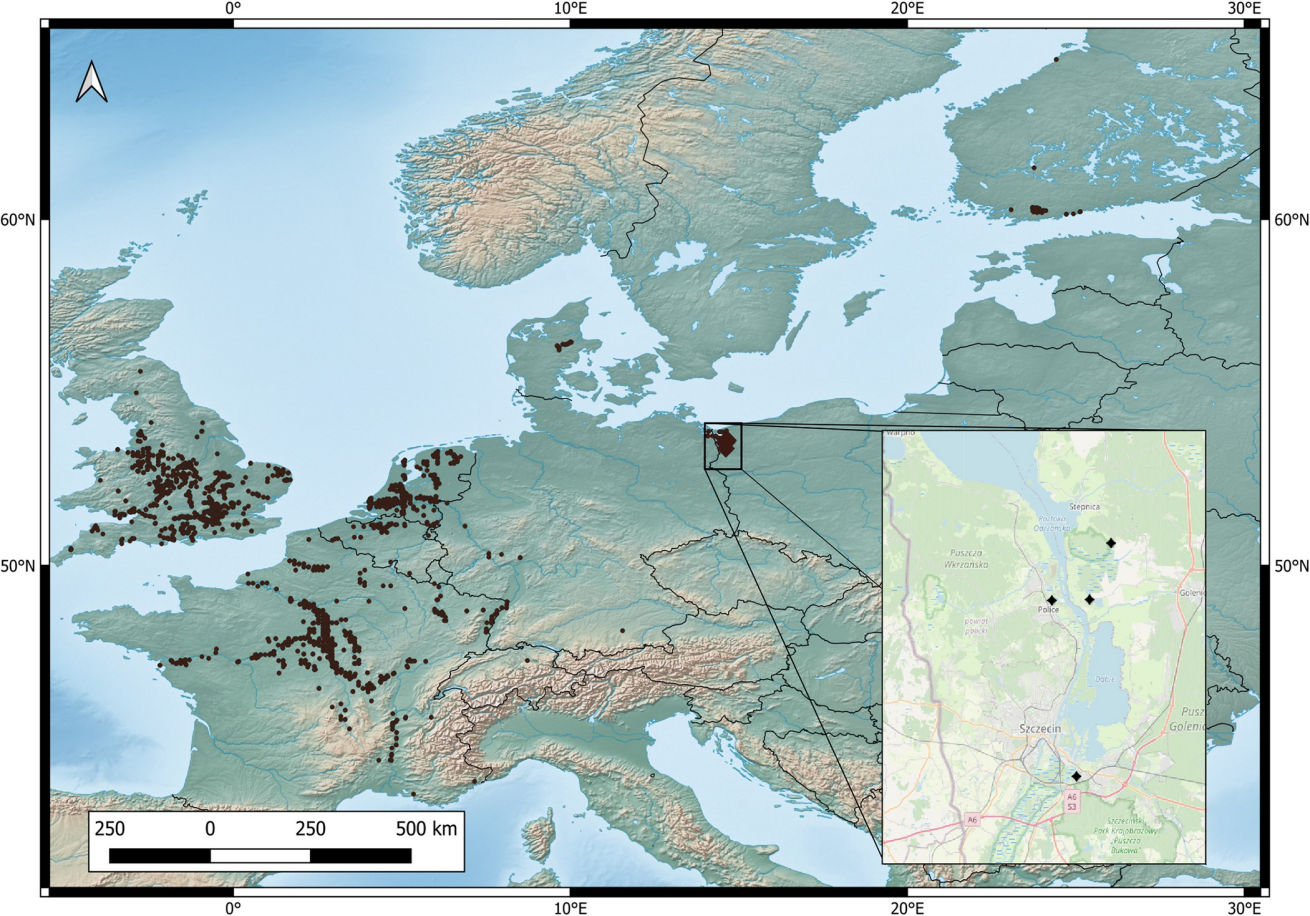
Map of European populations of *Impatiens capensis* (black dots), and populations sampled in Poland (black diamonds). Map generated with the courtesy of Natural Earth and modified in QGIS 3.20.2 (public domain).

**Fig 2:**
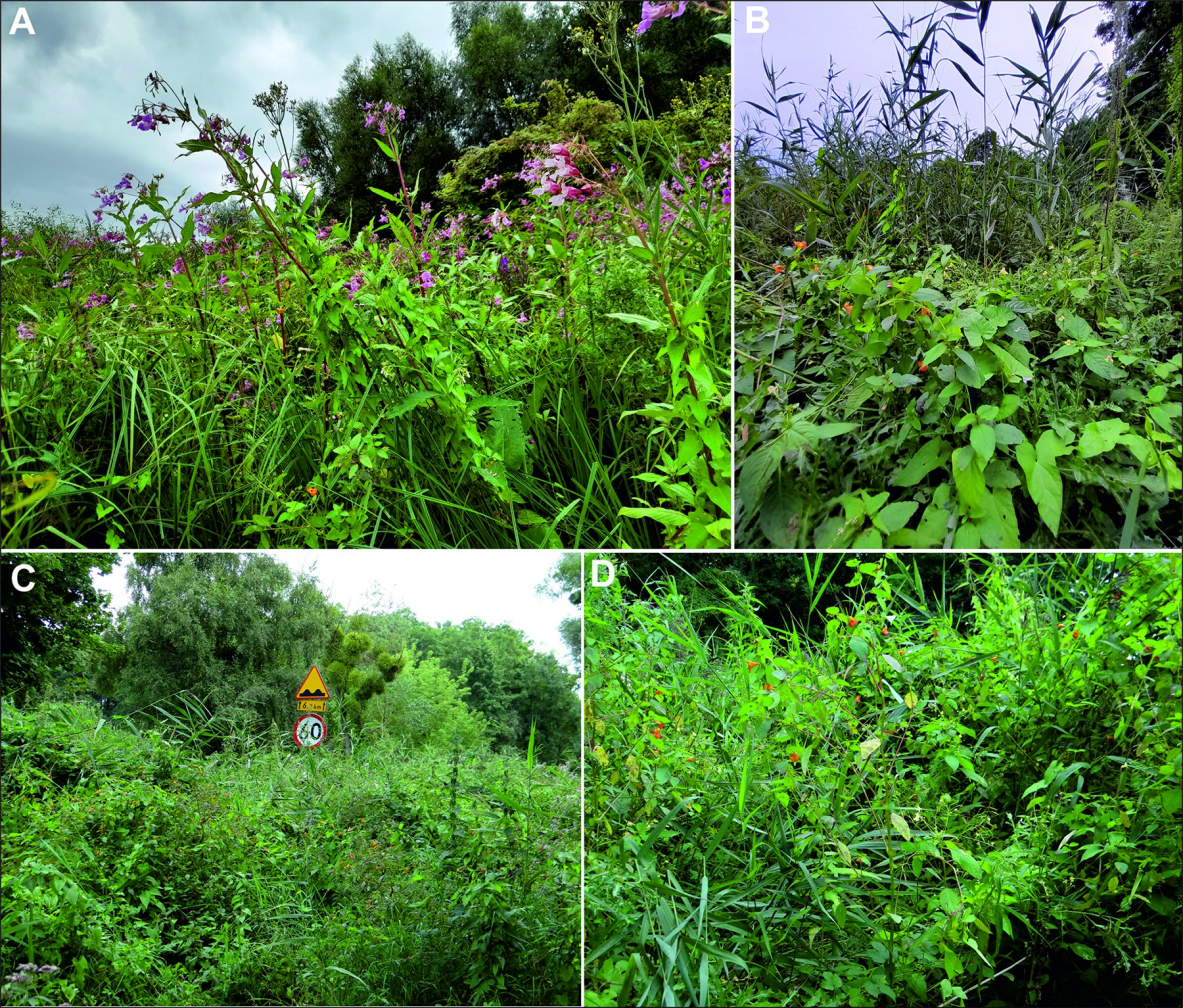
General view of the research sites in Police (A), Szczecin-Zdroje (B), Święta (C), and Budzień (D) (photo A. Rewicz 2021).

Four populations of *Impatiens capensis* were selected in the North West of Poland: (see Fig 1): Police (53.55788, 14.57061)—tall herbaceous ruderal plant community with dominance of *Impatiens glandulifera* and participation of *I*. *capensis*; Szczecin-Zdroje (53.382861, 14.614944)—natural plant community with the participation of *Epilobium hirsutum* L., *Urtica dioica* L., *Galeopsis bifida* Boenn., *Lycopus europaeus* L., *Calystegia sepium* (L.) R. Br., *Phragmites australis* (Cav.) Steud., *Phalaris arundinacea* L., *I*. *parviflora* and others; Święta (53.559442, 14.638376)—natural dense tall herbs along the provincial road, on the edge of a peat-covered meadow, with a network of drainage ditches, with the participation of *C*. *sepium*, *Carduus crispus* L., *Galeopsis speciosa* Mill., *Cirsium oleraceum* (L.) Scop., *U*. *dioica*, *Myosoton aquaticum* (L.) Moench, *Sonchus asper* (L.) Hill and others; Budzień (53.6152111, 14.67594)—natural tall herb fringe community, at the junction of roadside willow thickets and a damp mowed meadow, with the participation of *Impatiens parviflora*, *U*. *dioica*, *Cirsium arvense* (L.) Scop., *P*. *australis*, *Poa trivialis* L. and others (Figs 1 and 2).

#### Flower visitors

Insects were caught from the fresh flowers in August 2021 using entomological hand nets by two-person teams between 10 a.m. and 7 p.m. (for three days per population). Insects were collected from 5–7 plants growing close to each other. The material was collected when the transfer of pollen by insects was observed. Visitors of *I*. *capensis* were divided into two types [49, 76]: a) pollinators—invertebrates that either tried or successfully collected pollen from flower, b) nectar robber—invertebrates gnawing at the flower spur to obtain nectar, or feeding on the nectar (like pollinator), but without touching pollen or stigma because of the small size [76]. Captured invertebrates were preserved in 95% ethanol for further taxonomic identification using standard morphological keys [77, 78] and molecular methods. Nomenclature of insect mouthparts was provided according to Gillott [77], and after Jackiewicz [79] for snails.

#### Molecular identification through DNA barcodes

The DNA was extracted from a single leg, or piece of tissue (according to the animal´s size) using the standard Chelex method [80]. The standard gene region for animal DNA barcoding (COI -cytochrome c oxidase subunit I), as described by Hebert et al., [60], was amplified during the Polymerase Chain Reaction (PCR), using the primers LCO1490-JJ (5′-CHACWAAYCATAAAGATATYGG-3′) and HCO2198-JJ (5′-AWACTTCVGGRTGVCCAAARAATCA-3′) [81], under the following PCR conditions: 60 s at 94°C, 5x (30 s at 94°C, 90 s at 45°C, 60 s at 72°C), 35x (30 s at 94°C, 90 s at 51°C, 60 s at 72°C), 5 min at 72°C, as proposed by Hou et al. [82]. The PCR products (5 μl) were purified using Exonuclease I (20 U/μl, EURx, Poland) and alkaline phosphatase (Fast Polar-BAP 1 U/μl, EURx, Poland) following the manufacturer’s instructions. These purified products were sequenced at Macrogen Europe (Netherlands). The obtained sequences were edited and trimmed from primers using Geneious 10.2 [83]. Sequences were checked to verify the presence of obvious contaminations using BLAST [84] and were deposited in GenBank under accession numbers (OM794624—OM794682). Moreover, to obtain the Barcode Index Numbers (BIN) clustering together similar DNA sequences based on the genetic distance, as tentative equivalents of species [85], the sequences were deposited in the Barcode of Life Data Systems (BOLD; http://v4.boldsystems.org [86]) under Process IDs (POLIN001-21—POLIN070-21). Each specimen was identified by comparing the sequence assigned to the particular BIN and checking the deposited sequences in BOLD. Moreover, we execute the Batch ID engine upon a complete COI database with a 98% similarity threshold. Relevant voucher information, taxonomic classification, photos, and DNA barcode sequences are publicly accessible through the dataset DS-POLICAP (dx.doi.org/10.5883/DS-POLICAP) in BOLD.

### Functional traits

First, a list of pollinator species from the native range of *I*. *capensis* was compiled (S1 Table for references). Three traits were selected for the functionality of the pollinators assemblages: the status of the pollinator (i.e., true pollinator or robber/thief), its mouthparts composition (i.e., biting, chewing, sponging, licking, or mixed chewing + sucking, and piercing + sucking), and its body size [78, 87–92]. Six species were excluded from the analyses for the paucity of data or because of highly different traits: the hummingbird *Archilochus colubris* as it was the only vertebrate, four lepidopterans (i.e., *Battus philenor*, *Danaus plexippus*, *Papilio troilus* and *Phyciodes tharos*) because the size of their wings and their proboscis are too divergent as traits, and the hymenopteran *Augochlora* spp. because another species of this genus was already present and was a single record. Abrol [93] categorized animals from diverse groups as pollinators, including invertebrates such as snails and birds and mammals like bats. The analysis of collected materials in the context of the type of food consumed (consistency) and the type of mouthparts necessitated categorizing snails as insects according to the food source. Taking into consideration all zoological factors, it was determined that the feeding method of the snail *Succinea putris* is functionally closest to the chewing-sucking (CS) mouthparts, which are characteristic of hymenopterans in the family Apidae. Information about the three traits was compiled from specialized literature, experts’ opinion, or direct observation (i.e., for specimens found in this study). Fuzzy coding [94] was used to assign, to every taxon from our study and the literature (S1 Table), scores to the modalities of the three functional traits (Table 1) [95] (i.e., assigning affinity values from 0 to 3, with 0 meaning no affinity and 3 highest affinity).

**Table 1:** Functional traits selected for *I*. *capensis* visitors (pollinators and robbers).

Trait	Modalities	Description
Status (ST)	ST.P	Pollinators
	ST.T	Robbers/Thieves
Mouthparts (MP)	MP.B	Biting
	MP.C	Chewing
	MP.S	Sponging
	MP.L	Licking
	MP.CS	Chewing + Sucking
	MP.PS	Piercing + Sucking
Size in mm (SI)	SI.1	< = 7
	SI.2	7 < = 10
	SI.3	10 < = 15
	SI.4	>15

### Data analysis

For each species recorded in this study, its relative frequency of occurrence [96] was calculated [97]. Frequency (Fi) was calculated according to the formula: Fi = (j_i_ / k) × 100%, where: j–the number of places, in which species ‘i’ was recorded, and k–the total number of places. Bipartite graphs were done between species and sites with the package “bipartite_2.18” [98] of the software R version 4.3.0 [99].

To investigate the functional diversity of the pollinators, using Gower distances between species, the contribution of each trait was calculated (function *kdist*.*cor*). Additionally, we performed with the same distance matrix a fuzzy principal component analysis (FPCA) utilizing the *dudi*.*fpca* function. These analyses were carried out using the “ade4_1.7–22” package [100], and the results were visualized with the “factoextra_1.0.7” package [101] in R version 4.3.0 [99].

## Results

**Fig 3:**
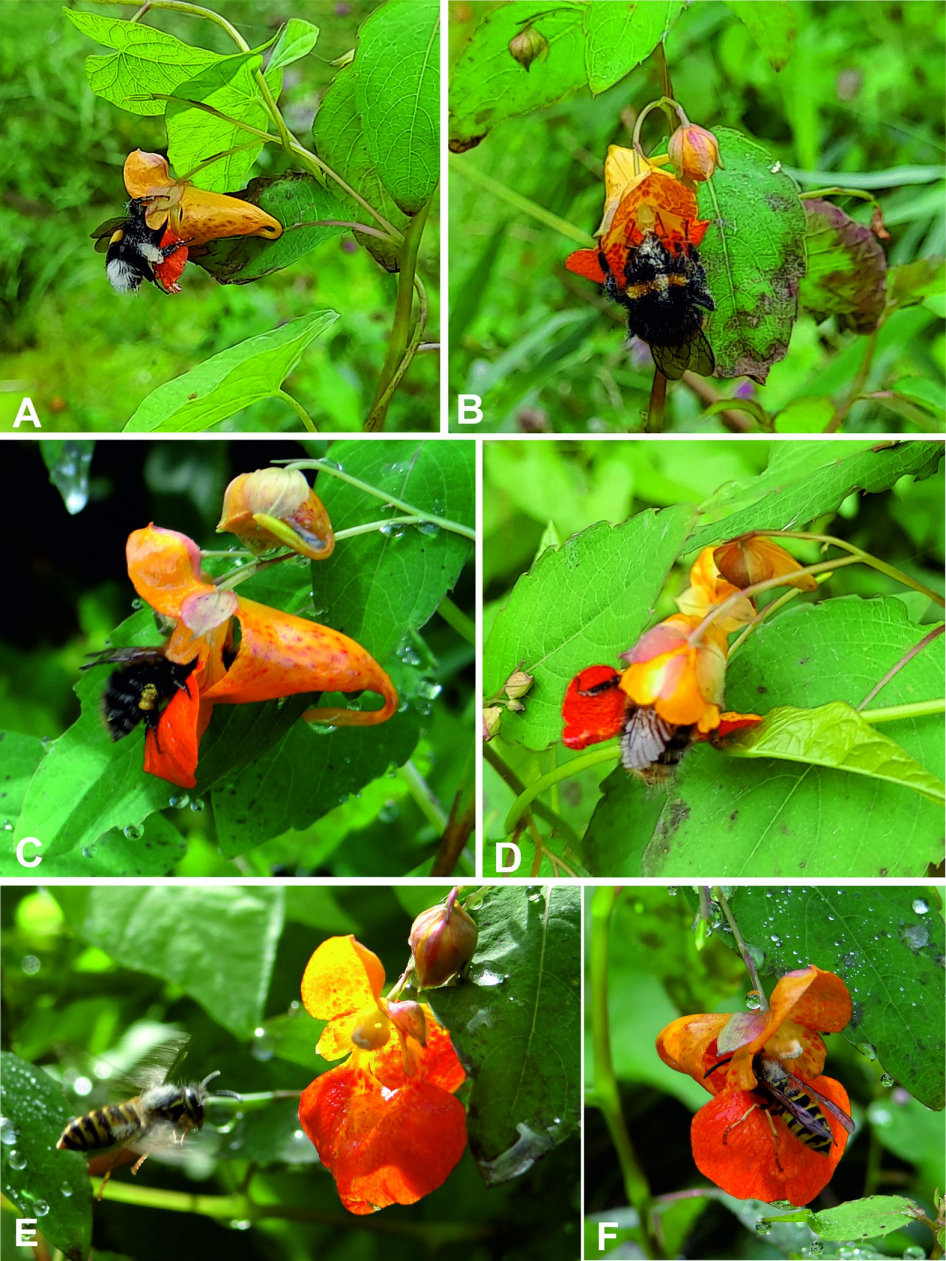
Pollinators of *Impatiens capensis* (photo A. Rewicz 2021): A-D bumblebees (Apidae), E, F—wasps (Vespidae).

**Fig 4:**
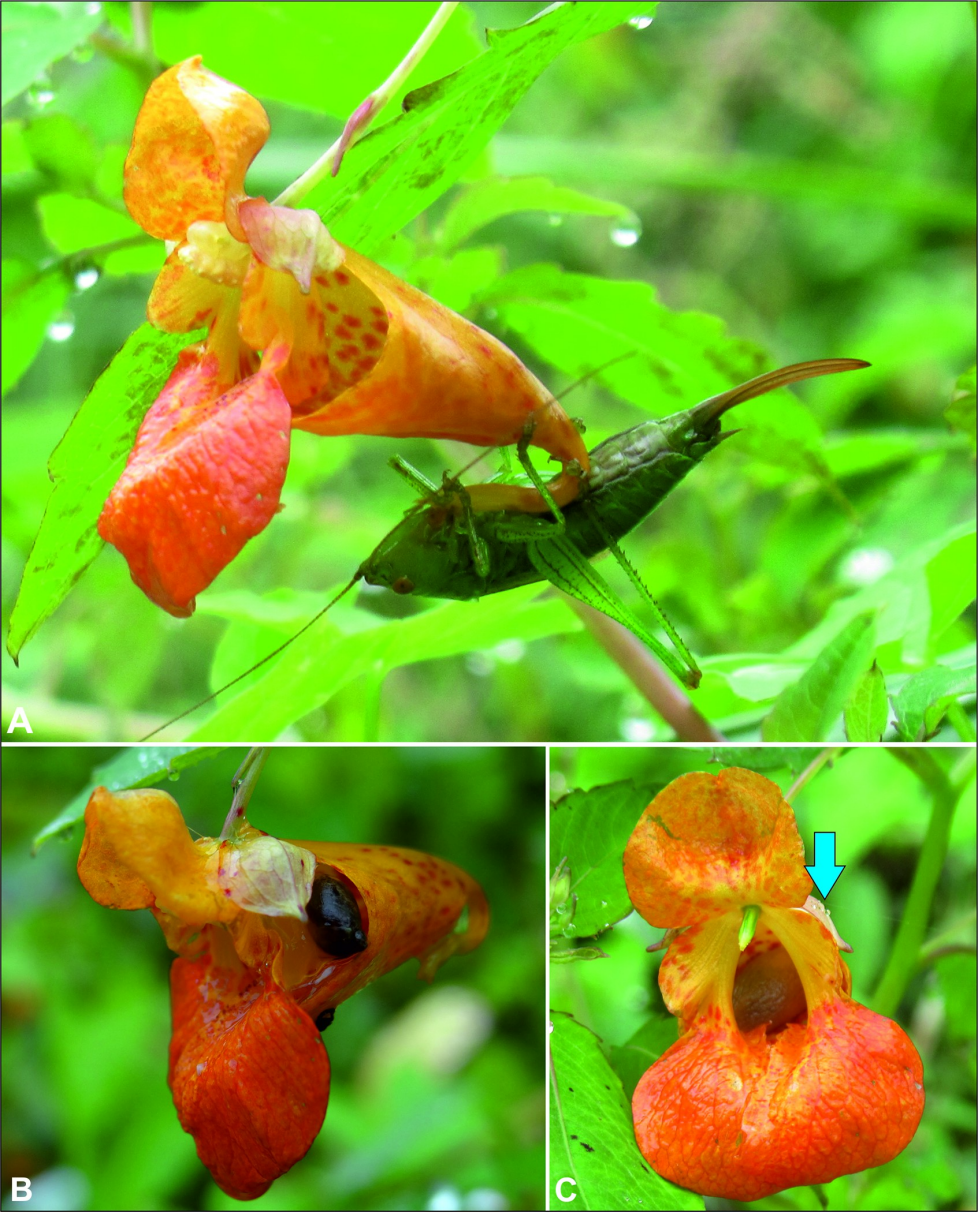
Nectar robbers of *Impatiens capensis* (photo A. Rewicz 2021): A—*Leptophyes punctatissima*; B, C—*Succinea putris*, explanation: the blue arrow points to the snail.

**Fig 5:**
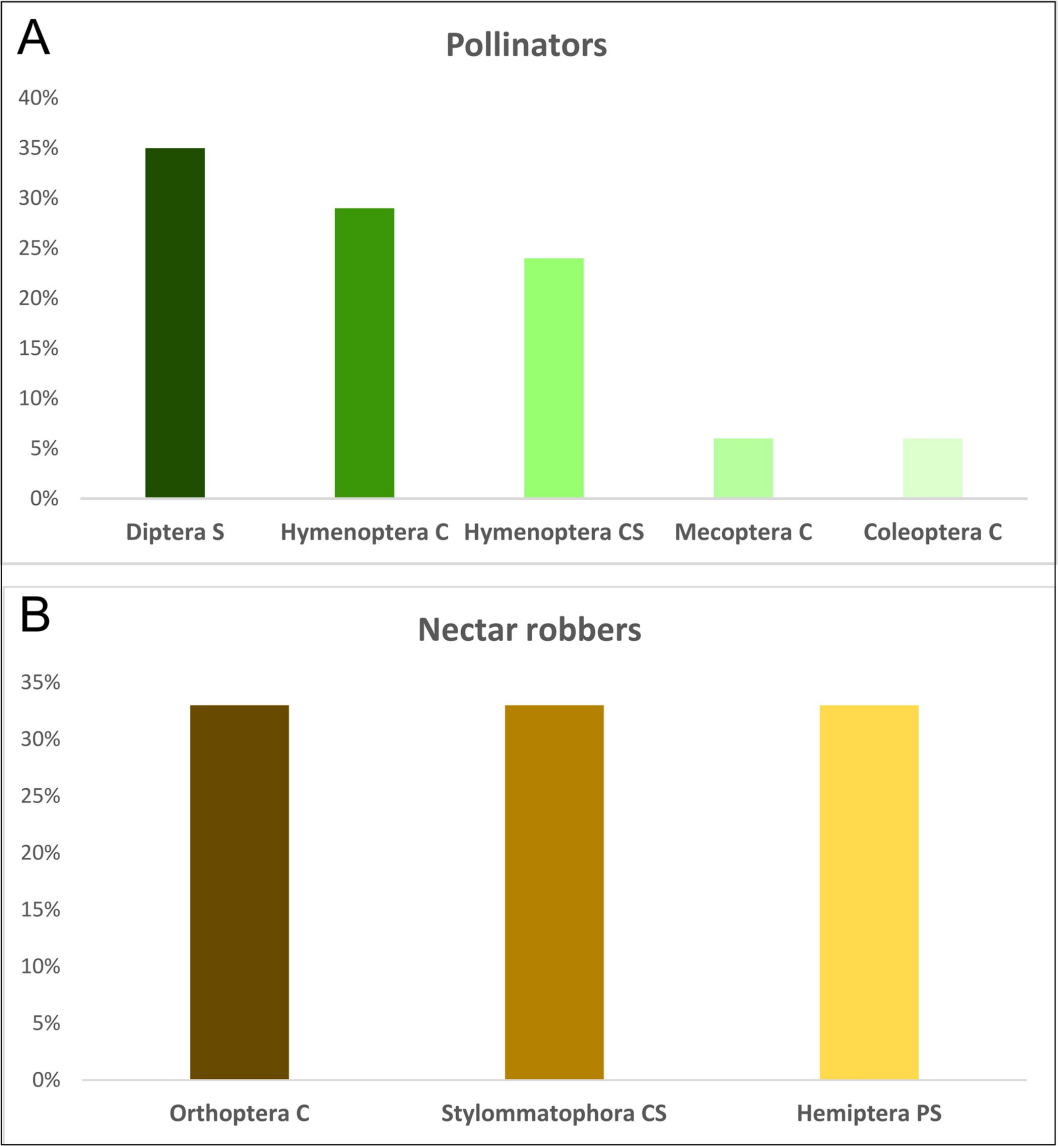
Diversity of *Impatiens capensis* pollinators and nectar robbers based on insect mouthparts (number of species). Explanations: C—chewing; S—sponging; PS—piercing and sucking; CS—chewing and sucking.

**Fig 6:**
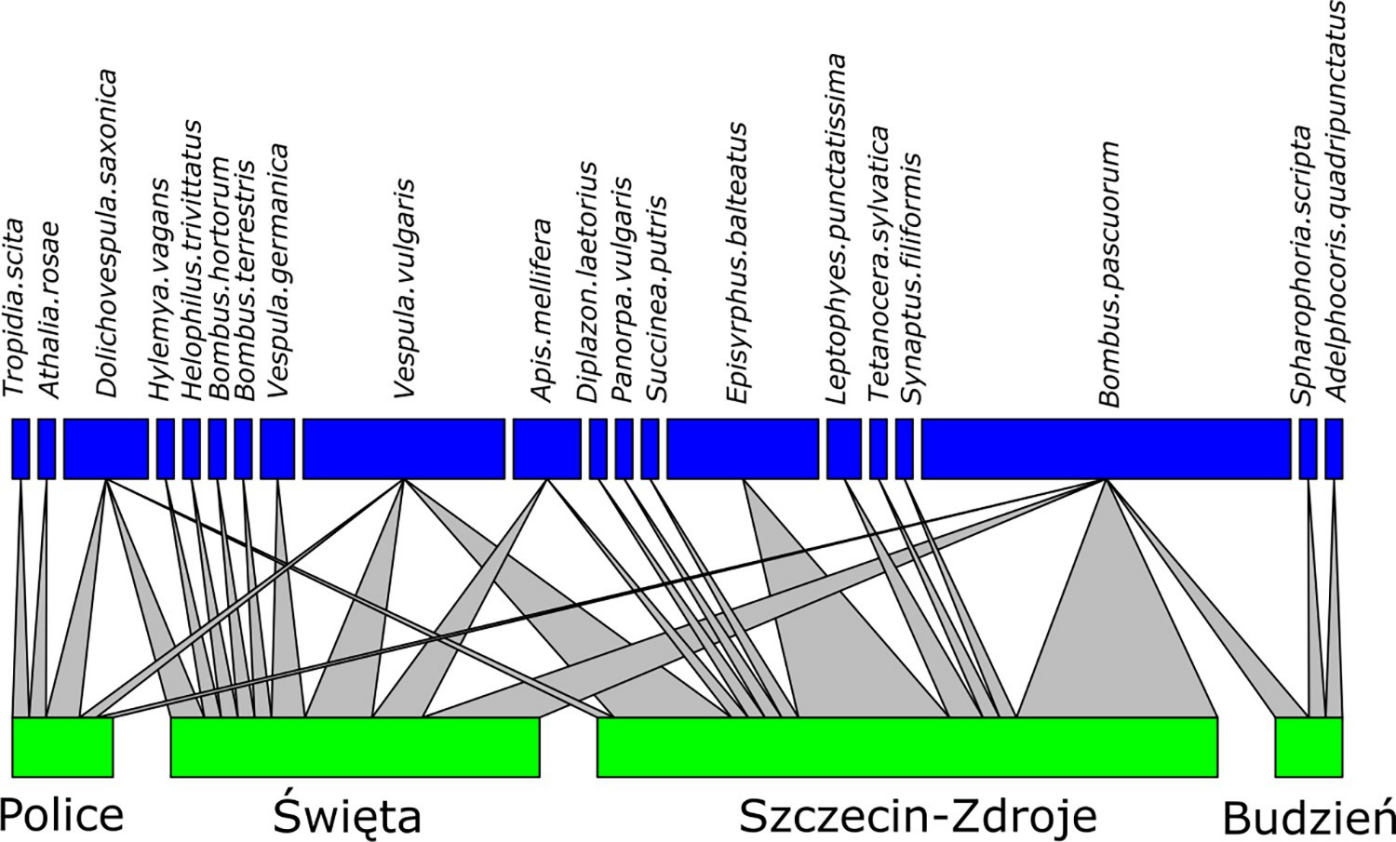
Bipartite graph between samples of different sites (green boxes) and species (blue boxes). Grey-filled triangles indicate each species’ relative presence and abundance in each site and the proportion of each species within each assemblage. Size of boxes is proportional to species abundance.

We collected a total of 69 invertebrate specimens. All of them were successfully identified at the species level using morphological features. Pollinators and nectar robbers of *Impatiens capensis* collected during this study belong to 20 species, 7 orders, and 12 families of invertebrates (S2 Table and Figs 3 and 4). Moreover, for the 59 individuals, we obtained COI barcodes assigned to 18 BINs. All BINs represent species occurring in Poland and matched with at least a 99% similarity threshold (Batch ID Engine, BOLD) to already deposited sequences. We detected no cryptic species/lineages, and none of the BINs were unique. Specimens from Hymenoptera and Diptera (75% and 21% respectively), dominated among the pollinating insects (excluding nectar robbers). Occasionally, single individuals of *Synaptus filiformis* (Coleoptera) and *Panorpa vulgaris* (Mecoptera) were also noted as pollinators of *I*. *capensis* (S2 Table). The group of nectar robbers included insects representing orders: Hemiptera (*Adelphocoris quadripunctatus*), Orthoptera (*Leptophyes punctatissima*), and snail: Stylommatophora (*Succinea putris*) (Fig 4). The main pollinators were: *Bombus pascuorum* (Hymenoptera) - 33.8% of all noted pollinators, with Fi = 100% recorded in all four places; *Vespula vulgaris* (Hymenoptera) - 18.5% and Fi = 75% recorded in three places; *Episyrphus balteatus* (Diptera) - 13.8% and Fi = 25% recorded in one place; *Dolichovespula saxonica* (Hymenoptera) - 7.7% and Fi = 75% recorded in three places (S2 Table and Figs 5 and 6).

According to the type of mouthparts, the main pollinators of *I*. *capensis* can be divided into three groups, according to species number: C, chewing insects—Coleoptera—6% of all noted pollinators, Hymenoptera—29% and Mecoptera—6%, S, sponging insects—Diptera—35% and CS, chewing and sucking—Hymenoptera 24% (Fig 5). In the case of nectar robber, three species with three different mouthparts were recorded (C—chewing; PS—piercing and sucking; CS—chewing and sucking, S2 Table).

**Fig 7:**
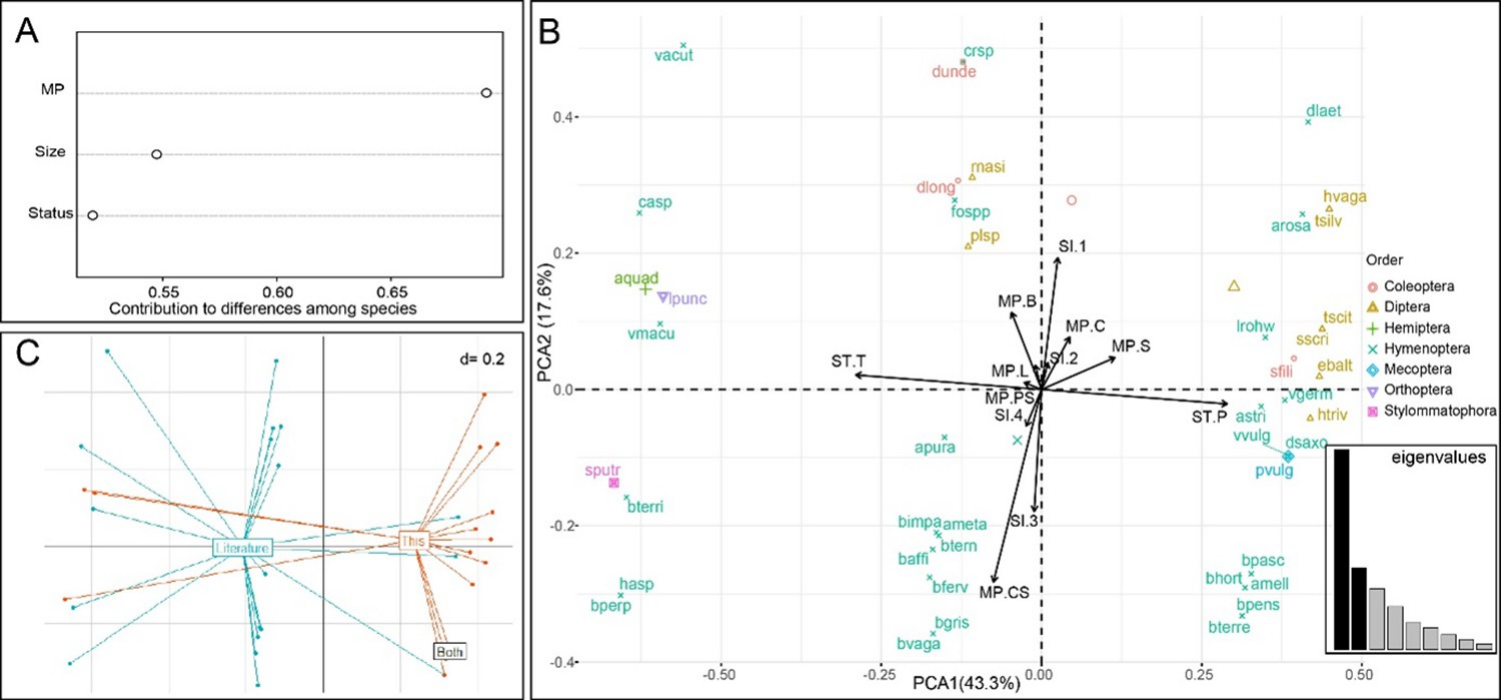
Contribution of each trait to the global distance of the functional traits for the insect pollinators (A), fuzzy principal component analysis plot (FPCA, with centroids of the orders displayed by the relative empty bigger shapes) (B), and scatter diagram (C) of the FPCA showing the centroids relative to the origin of the records (i.e., Literature = from native range, This = from this study, Both = from either native range or this study–only *Apis mellifera*). Extended names of the species in S3 Table. Colors in B refer to the different orders of the species. Traits explanation in Table 1.

The trait contributing in the highest proportion to the total functional variation among the pollinators was the mouthparts composition (Fig 7A). Nevertheless, the two modalities of the status (i.e., pollinators–ST.P- and robbers–ST.T) explained most of the variability in the first component of the FPCA (i.e., ~43%; Fig 7B).

The first component also discriminated almost completely the two assemblages either from the native range or in this study (Fig 7C). In fact, only 15% of the species detected in this study was strictly a robber (i.e., *Adelphocoris quadripunctatus*, *Leptophyes punctatissima*, *Succinea putris*), while more than 50% (i.e., 20 out of 38 species in total) of the species from the native range were reported as strict or occasional robbers (S2 Table). The second component was explained principally by the chewing + sucking mouthparts and the size classes, with bigger animals primarily localized in the lower quadrants and smaller ones in the opposite.

## Discussion

### Pollinators in the native and invasive range of *I*. *capensis*

Sixty-eight species in *I*. *capensis* were identified from 50 published sources from 1672 to 2023, with 44 papers carrying out research in the USA and the rest in Canada. The visitors presented five categories of behaviour: pollinators, nectar thieves/robbers, plant consumers, parasites, and predators of pollinators. However, several works did not categorize the behavior of the visitors, while in others, the identifications were limited to the genus level or generalized to familiar names (see S1 Table).

In this study, we observed that in the established Polish populations of *Impatiens capensis*, there are several congeneric pollinator species similar to those in the native populations from North America. However, these pollinators differ from the native ones in terms of their mouthparts characteristics and their use of this plant, displaying generally smaller sizes and an absence of vertebrates (i.e., hummingbirds). Hymenoptera pollinators represent the highest widespread species richness (nine spp.), *Bombus pascuorum* being the most frequent visitor, presenting a 100% of prevalence in all sites, followed by *Dolichovespula saxonica* and *Vespula vulgaris* (75%) and *Apis mellifera* (50%). This is in accordance with numerous studies from different European regions that have shown that *Impatiens* species’ main pollinators are mostly insects from Hymenoptera orders. For example, *I*. *noli-tangere* has been found to be pollinated by species such as *Bombus lapidarius*, *B*. *hortorum*, *B*. *terrestris*, *Halictus cylindricus*, and *H*. *zonulus* [102]. Similarly, *I*. *glandulifera* and *I*. *balfourii* have been observed to be pollinated by *Apis mellifera*, *B*. *hortorum*, and *B*. *pascuorum* [103]. Similar patterns in the frequent visits of Hymenopteran pollinators are reported in North American native and introduced populations of *I*. *capensis*, especially by the frequent *A*. *mellifera* (see S2 Table, supplementary materials), which is known for its efficient pollination capabilities across many plant species worldwide [104, 105]. European and American populations present other visitors of *Apis*, *Bombus*, and *Vespula* genera, with Polish populations of this study exhibiting visits from the widespread pollinators *B*. *hortorum*, *B*. *pascuorum*, *V*. *germanica* and *V*. *vulgaris* (see S2 Table). On the other hand, we see congeneric species, such as endemic species like *B*. *fervidus*, *B*. *impatiens*, *B*. *vagans*, *V*. *maculifrons* (see S2 and S3 Tables) are the primary North American pollinator visitors, with occasional reports of other pollinators such as *B*. *affinis* [49], *B*. *griseocollis* [106, 107], *B*. *pennsylvanicus* [107] and *B*. *perplexus* [108].

These changes in genera and species between continents may reflect principal mechanisms of pollination effectiveness with particular emphasis on species of Apidae and Vespidae families, designs that are kept despite geographical barriers, it is suggested in native and non-native *Impatiens* no remarkable changes in functional groups of pollinators [38] suggesting a morphological fit between pollinator-flower. For example, native populations of *I*. *edgeworthii* in India [109] and non-native populations in Europe [110] share similar functional groups composed of species of honeybees (*Apis* sp.) and bumblebees (*Bombus* spp.). Similarly, several exotic populations of *I*. *glandulifera* found in America [111, 112] and Europe [113–115] share a pattern of frequent pollinator visitors principally from species of the genus *Bombus*, the honeybee (*Apis mellifera*) and the wasp (*Vespula vulgaris*). While in native populations of *I*. *noli-tangere* in Europe [9, 115] and Japan [116] occur principally several different species of bumblebees (Europe: *B*. *pascuorum*, *B*. *hortorum*, *B*. *lapidarius*, *B*. *wurflenii*, *B*. *terrestris*; Asia: *B*. *diversus*, *B*. *honshuensis*, *B*. *hypocrita*, *B*. *usurrensis*). Moreover, other genera of pollinator visitors are documented in *I*. *capensis* for each continent; in European populations of the present investigation, sawflies *Athalia rosae*, and wasps *Diplazon laetatorius*, and *Dolichovespula saxonica* so long as in American populations, endemic bee *Lasioglosum rohweri* [117, 118], sweet bees; *Augochlora* spp. [119], *Augochlorella striata* [120], *Augochlorella* spp. [119] and the widespread ant *Camponotus* sp. [49]. The occurrence of Hymenoptera order visitors, which is common in both non-native and native regions, indicates the presence of the bee syndrome in the orange balsam.

The results of the FPCA show potential trait combinations that primarily facilitate pollination, such as the ability to chew and suck, and larger body size (between 10 and 15 mm). In addition, another functional group was identified for the European populations, displaying smaller body size (less than or equal to 7 mm) and suction capacity. Rust [49], linked specific morphological traits of visitors with pollination effectiveness in *I*. *capensis*, e.g. thoracic height in various visitors was found to be associated with pollination effectiveness, irrespective of their body sizes or mouthpart types. In fact, some organisms are favored in obtaining nectar and are effective pollinators, as is the case of the hummingbird *Archilochus colubris* (not included in the functional analyses) in American populations presenting a long beak with retractile tongue favoring robbing deep saccate sepals, and a culmen that can touch the stamens [98]. Similarly, some European species have analogous traits like the long proboscis of like *Macroglossum stellatarum* specialized in stealing nectar also from *Impatiens* species (*I*. *capensis*: T. Rewicz personal observation, S1 Video and S1 Fig; *I*. *glandulifera*: see [121].

We also detected other critical pathways to success related to pollination systems (flower-visitor morphological traits) and foraging behavior. Our results show mainly nectar foraging within the saccate sepal, while pollen foraging to a lesser degree. To obtain nectar from the flowers, *Bombus* and *Vespula* enter the body part to the removed sepal, resting the back legs on the lateral sepals while the dorsal face of the thorax touches the pollen (see Fig 3C and 3F). In fact, morphological traits such as the lateral petals with support function and the disposition of the sexual structures represent essential factors in the impregnation of pollen in the dorsal region of the visitors, similar studies by Rust [49, 122] and Wilson & Thomson [118] evidenced the same form of entry and pollen impregnation in the *B*. *vagans* bumblebee. Buening [123] conducted studies that documented two entry forms of the bees: one involves them directly landing on the sepal in a saccate manner, while the other involves them crawling inside the structure. Regardless of the mode of entry, pollen impregnation occurs on the bees’ back. *Bombus* species, despite being nectar-collecting bees [124], also engage in pollen foraging (Fig 3B). During our observations, we noticed specimens of this genus adopting a behavior where they land on the flower and embrace it by pinning their back legs on the distal lobe while grasping the basal lobe and lateral sepals with their anterior legs. This behavior has been reported in other studies as well. This type of double foraging favors pollen impregnation on the mouth, dorsal, and caudal regions of the bumblebees. In this study, specimens of *Bombus* showed pollen impregnation also on the back limbs, while some of *Vespula* genus on the lateral part of the abdomen (see Fig 3A, 3B, 3D and 3E).

In our research, we observed that other attributes associated with the frequency of pollinator visits include perianth color and morphological characteristics of the flower. For instance, the flowers we studied were orange in color and had lateral petals overlapping each other. Additionally, they were connected to an elongated saccate sepal, which ended in a recurved and long spur. These specific flower traits may be the reason why they are mainly visited by Hymenopteran species, particularly representatives of *Bombus* (Figs 3 and 4) [5, 12]. Ruchisansakun et al. [38] associated the attributes of a large floral entrance and a short spur with the "bee-pollination syndrome". Moreover, the pink and yellow to orange color of the perianth were associated with more bee visits. Variants of *I*. *capensis* of the present work (see Figs 3 and 4) present a recurved (180°) spur that is used by other visitors (especially grasshoppers robbers) as a support structure to obtain nectar (see similar patterns in Rust [49]). In studies by Young [125], curved spurs have also been associated with the increased presence of robbers.

It has been documented that flowers may present adaptations associated with effective pollinators or groups of affined pollinators known as pollinator assemblages [38, 126]. However, the visits of other pollinators (e.g. secondary pollinators) do not always encompass the proper characteristics of the syndrome, Effective secondary pollinators with different morphological structures were reported in *I*. *capensis* in European and American populations; similarly, studies by Vlasankova et al. [105] in *Impatiens burtonii* demonstrate that under-expected visitors (long-proboscis) adapted to large corollas and spurs are likewise visited by members with contrasting characteristics (short-proboscis) and being efficient pollinators, suggesting that those morphological characteristics in the flower are more generalized to the visitors.

### The case of robbers and morphological traits that allow robbery

Few robbers were reported in the European populations of *I*. *capensis*, which were categorized as "strict robbers" since they did not contribute to pollination and obtained nectar directly from flower borers. Our research is also in line with this trend, out of the pool of insects we recorded, only three species were classified as robbers. The total number of individuals was four, of which only *Leptophyes punctatissima* was recorded twice on flowers with over 60 specimens of insects classified as pollinators. However, in the case of populations from North America, there is a remarkable variety of organisms that act as strict or occasional robbers (when carrying out pollination processes as well; see S2 Table). In contrast, some authors categorize visiting species as primary and secondary robbers [48, 49] since they make holes in the flower, while others only take advantage of previous holes for feeding. Furthermore, there are small organisms with the ability to enter through the saccate sepal without touching the floral structures (e.g., *Augochlora* spp., *Crematogaster* sp., *Formica* spp. [49, 127]), but this was not the case for European robbers due to their size (*L*. *punctatissima*) that have other characteristics, such as biting-boring or drilling, but above all sucking (*Adelphocoris quadripunctatus*, *Succinea putris* and *Leptophyes punctatissima*) that facilitate this type of foraging. However, some probable morphological characteristics favoring stealing success are reminiscent of the anchoring-adherence capacity of visitors to flower structures such as the spur (anterior and posterior legs in *L*. *punctatissima*, Fig 3A), or on the saccate sepal (adhesion capacity in *S*. *putris*, Figs 4B and 4C).

## Conclusions

For the first time, we present information on visitors to *Impatiens capensis* in a non-native area and compare them with the available data from populations in the U.S.A. and Canada. The involvement of pollination agents can help us understand the spread and success of the orange balsam. The frequency of visitors from the Hymenoptera order, common in both non-native and native areas, suggests the presence of the bee syndrome in the orange balsam. Our research reveals the identification of nine species within the Hymenoptera group. Among these, four species exhibited a frequency ranging from 50% to 100% in the surveyed sites of *I*. *capensis*. This observation is supported by the flower’s morphological characteristics. Through this syndrome, the species efficiently carries out the pollination process, attracting various primary pollinators such as bees, bumblebees, and wasps. Moreover, it establishes mutualistic associations with secondary pollinators like Diptera and Lepidoptera, which further enhances the probability of successful pollination. Larger scale studies on the pollination biology of *I*. *capensis* supported by molecular tools, may provide useful information about the pollination success of this widespread species and its visitors, as well as provide an understanding of a co-evolutionary process likely present.

## Supporting information

S1 Fig *Macroglossum stellatarum*—pollinator of *Impatiens capensis* (photo T. Rewicz 2022). (TIF)

S1 Table Visitor records (pollinators, nectar robbers/thieves, plant consumers, parasites and pollinators predators inhabiting organisms of *I*. *capensis*). (DOCX)

S2 Table Pollinators and nectar robbers of *Impatiens capensis* noticed in Poland. (DOCX)

S3 Table Fuzzy coded matrix. (DOCX)

S1 Video *Macroglossum stellatarum*—pollinator of *Impatiens capensis* (video T. Rewicz 2022). (MP4)
