# Supplementary figures and images for: Pollination biology of Impatiens capensis Meerb. in non-native range
Source: PLoS One. 2024 Jun 20;19(6):e0302283. doi: 10.1371/journal.pone.0302283 (PMC11189253; doi:10.1371/journal.pone.0302283)

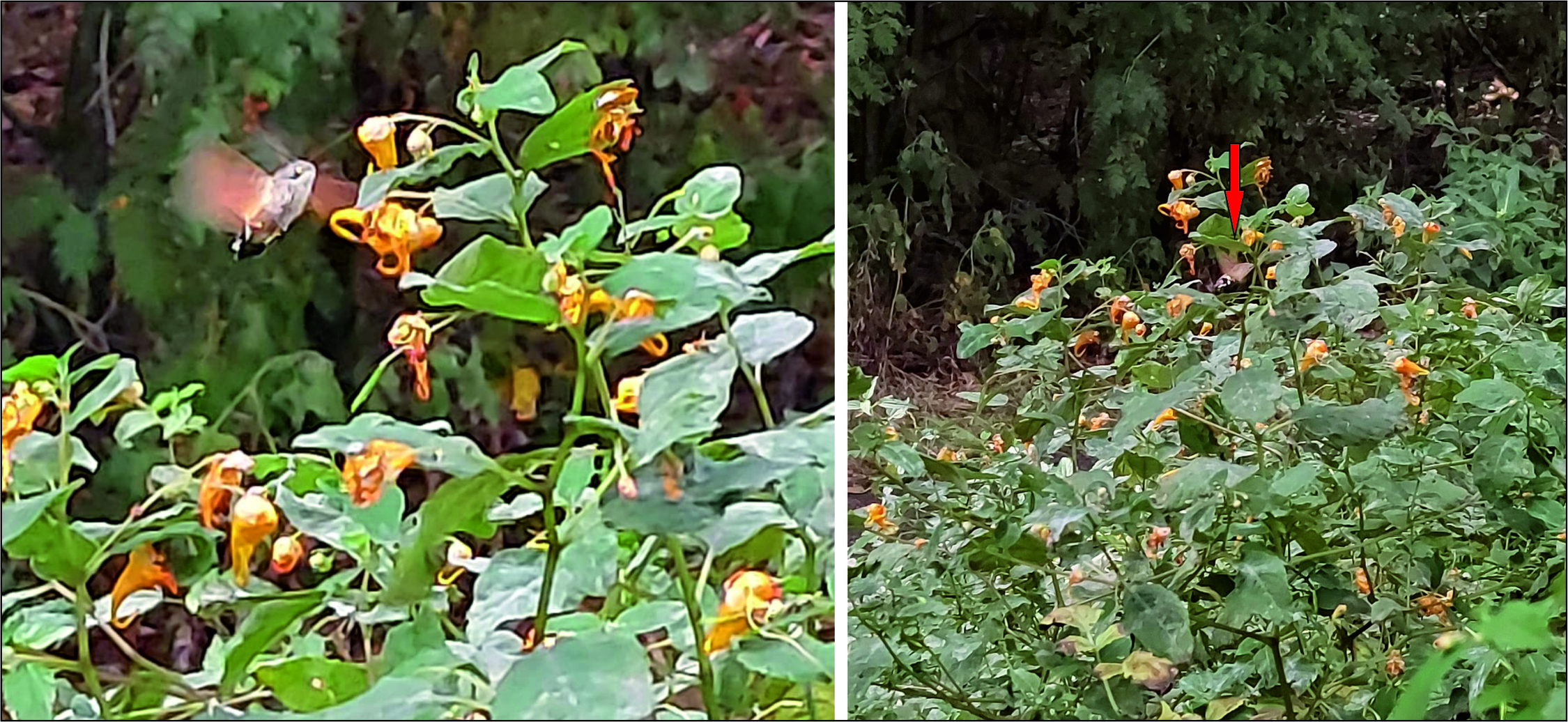

Supplement: S1 Fig — (TIF) [file pone.0302283.s001.tif]
