# Supplementary material for: Pollination biology of Impatiens capensis Meerb. in non-native range
Source: PLoS One. 2024 Jun 20;19(6):e0302283. doi: 10.1371/journal.pone.0302283 (PMC11189253; doi:10.1371/journal.pone.0302283)
Supplement: S1 Table — (DOCX) [file pone.0302283.s002.docx]

S1 Table. Visitor records (pollinators, nectar robbers/thieves, plant consumers, parasites and pollinators predators inhabiting organisms of *I. capensis*)

| **Visitors observations and records** | **Country** | **Reference** |
| --- | --- | --- |
| Hummingbird $ | U.S.A | [1] |
| Bees (Humblebees [bumblebees] **) | U.S.A | [2] |
| Birds (Hummingbirds ***)  Bees (Black bees **, honey bee **, humble bee **, small wild bees **) | U.S.A | [3] |
| Bees (hive bees [honey bees] **) | U.S.A | [4] |
| Birds (Hummingbirds **) | U.S.A | [5] |
| Bees (Bumblebees **, honey bees *) | U.S.A | [6] |
| Bees ***  Bees (Bumblebees *)  Flies **  Birds (Hummingbirds *)  Smaller bees $ | U.S.A | [7] |
| Flies (Leaf miner fly; *Agromyza borealis* Malloch, 1913 %) | U.S.A | [8] |
| Bees (Bumble bees; American bumblebee; *-Bombus pensylvanicus* De Geer, 1773 $*,* Common eastern bumble bee; *B. impatiens* Creeson, 1863 $, honey bees; *Apis* sp. $*,* leafcutting bees; *Megachile brevis* Say, 1863 $, Flat-tailed leaf-cutter bee; *M. mendica* Creeson, 1878 $, long-horned bees; Two-spotted longorn bees; *Melissodes bimaculata* Lepeletier, 1825 $, Experienced sweet bee; --*Lasioglossum versatum* Robertson, 1902 $, Golden sweet bee; *---Augochlorella aurata* Smith, 1853 $)  Beetles (Spotted cucumber beetle; *Diabrotica 12-punctata* Fabricius, 1775 $)  Birds (*Trochilus* sp*.* $)  Butterflies (*Euphoeades* sp. $) | U.S.A | [9] |
| Birds (Hummingbirds; Ruby throated; *Archilochus colubris* Linnaeus, 1858 ***) | U.S.A | [10] |
| Birds (Hummingbirds; Ruby throated; *Archilochus colubris* ***) | U.S.A | [11] |
| Birds (Hummingbirds; Ruby throated; *Archilochus colubris* $) | U.S.A | [12] |
| Mosquitoes (Gall midges**;** Jeweelded midge; *Schizomyia impatientis* Osten Sacken, 1862 %) | U.S.A | [13] |
| Bees (Bumblebees $)  Birds (Hummingbirds $) | Canada | [14] |
| Bees ***  Birds (Hummingbirds; Ruby throated; *Archilochus colubris* *)  Wasps ** | U.S.A | [15] |
| Bees (Bumblebees; Yellow bumble bee; *Bombus fervidus* Fabricius, 1798 ***,* Tricolored bumble bee; *B. ternarius* Say, 1837 **, Yellow banded bumble bee; *B. terricola* Kirby, 1837 **, Half-black bumble bee; *B. vagans* Smith, 1854 ****) | U.S.A | [16] |
| Bees (Bumblebees; Yellow bumble bee; *Bombus fervidus **,* Brown-belted bumblebee; *B. griseocollis* De Geer, 1773 ****, Tricolored bumble bee; *B. ternarius *** Say, 1837, Yellow banded bumble bee; *B. terricola ***, Half-black bumble bee; *B. vagans ***) | U.S.A | [17] |
| Birds (Hummingbirds; Ruby-throated; *Archilochus colubris **)  Bees (Honey bees; *Apis mellifera* *, bumblebees ***)  Diptera *  Hemiptera *  Hymenoptera * | U.S.A | [18] |
| Ants (*Crematogaster* sp*.*** [small thieves])  Bees (Honey bees; *Apis mellifera **** [secondary thief], bumblebees; Half-black bumble bee; *Bombus vagans ***,* Common eastern bumble bee; *B. impatiens *,* Rusty patched bumble bee; *Bombus affinis* Cresson, 1863 **** [principal thief], sweat bees; Metallic Epauletted-Sweat bee; *Augochloropsis metallica* Fabricius, 1763 ****, Pure golden green sweat bee; *Augochlora pura* Say, 1837 **** [secondary thieves, both robbing manners], *Lasiglossum* sp*. *** [small thieves])  Wasps (Eastern yellowjacket; *Vespula maculifrons* Buysson, 1905 ***** [primary thief]) | U.S.A | [19] |
| Aphids (Rufous jewelweed aphid;*- - - -* *Uroleucon impatiensicolens* Patch, 1919 $)  Bees (Bumblebees; Common eastern bumble bee; *Bombus impatiens **, Half-black bumble bee; *B. vagans **)  Beetles (Leaf beetles; *Rhabdopterus praetexus* Say, 1824 #)  Birds (Hummingbirds; Ruby throated*; Archilochus colubris **)  Bug (Green stink bug; - - - - - - -*Chinavia hilaris* Say, 1832 [nymphs] seed predator #)  Flies (Gall midges; - - - - - *Neolasioptera impatientifolia*  Felt, 1907 % , sawflies; *Aglaostigma semiluteum* Norton, 1862 #)  Mosquitoes (Gall midges; - - - - - - Jewelweed midge; *Schizomyia* *impatientis* Osten Sacken, 1862 %)  Moths (*Olethreutes agilana* Clemens, 1860 # [stem-boring larvae])  Wasps (Eastern yellowjacket; *Vespula maculifrons* $) | U.S.A | [20] |
| Bees (Honey bees; *Apis mellifera* ***, bumblebees; *Bombus* spp. *)  Beetles (Pumpkin beatles; *Diabrotica longicornis* Say, 1824 **, Western spotted cucumber beetle *Diabrotica* *undecimpunctata* Mannerheim, 1843 **)  Birds (Hummingbirds; Ruby throated*; Archilochus colubris **)  Leafhoppers (Cicadellidae $)  Syrphidae $ | U.S.A | [21] |
| Bees (Bumblebees *, honey bees *)  Birds (Hummingbirds; Ruby throated; *Archilochus colubris* *) | U.S.A | [22] |
| Ants (*Camponotus* sp. *** [primary robber and pollinator], *Crematogaster* sp. *** [secondary and small robber]  Bees (Honey bees; *Apis mellifera* ***, [secondary robber], bumblebees; Fork tailed flower bee; *Anthophora furcata* Panzer, 1978 * Rusty patched bumble bee; *Bombus affinis* *** [primary robber]), Common eastern bumble bee; *B. impatiens* *** [secondary robber], Yellow bumble bee; *B. fervidus* *, American bumblebee; *B. pennsylvanicus* *, Half-black bumblebee; *B. vagans* *** [secondary robber], sweat bees; pure gold-green sweat bee; *Augochlora pura* *** [secondary and small robber], Metallic Epauletted-Sweat bee; *Augochloropsis metallica* *** [secondary and small robber], *Lasioglossum cephalotes* Dalla Torre, 1896 *, *Lasioglossum forbesii* Robertson, 1890 *, *Lasioglossum* sp. *** [secondary and small robber])  Wasps (Common aerial yellowjacket; *Vespula arenaria* Fabricius, 1775 *, Eastern yellowjacket; *Vespula maculifrons* *** [primary robber], widow yellowjacket; *Vespula vidua* de Saussure, 1854 *) | U.S.A | [23] |
| Bees (Bumblebees; Common eastern bumble bee; *Bombus impatiens *,* Brown-belted bumblebee; *B. griseocollis* De Geer, 1773 *, sweat bees; Halictid bees *)  Birds (Hummingbirds; Ruby throat; *Archilochus colubris **)  Flies (Syrphid flies *) | U.S.A | [24] |
| Bees (Bumblebees; Confusing bumble bee; *Bombus perplexus* Cresson, 1863 *, Yellow banded bumble bee; *B. terricola* ***, *B. vagans *,* honey bees; *Apis mellifera* *)  Birds (Hummingbirds ***)  Ichneumonids $  Syrphids $  Wasps (+ *Vespula acutifrons* **) | Canada | [25] |
| Bees (Bumblebees; *B. fervidus* ***, Half-black bumble bee; *B. vagans* ***)  Birds (Hummingbirds; Ruby-throated; *Archilochus* spp. **) | Canada | [26] |
| Bees (Bumblebees; Half-black bumble bee; *Bombus vagans* **) | U.S.A | [27] |
| Bees (Bumblebees; Common eastern bumble bee; *Bombus impatiens* *, American bumblebee; *B. pennsylvanicus* *, Brown-belted bumblebee; *B. griseocollis* ***, honey bees; *Apis mellifera* *; miner bees; Andrenid *, sweat bees; *Halictus* sp. ***, leafcutting bees; megachilid bees *)  Beetles (Soldier beetles; Cantheridae *# [flower predator])  Birds (Hummingbirds; Ruby-throated * [secondary pollinator])  Wasps (Yellow jackets; *Vespula* spp. *** [secondary pollinator and nectar robber]) | U.S.A | [28] |
| Bees (Honey bees; *Apis mellifera* *, bumblebees; Half-black bumblebee; *Bombus vagans* *) | Canada | [29] |
| Bees (Honey bees; *Apis mellifera **, bumblebees; Common eastern bumble bee; *Bombus impatiens* ***, Half-black bumblebee; *B. vagans* ***, sweat bees; *- - - - - - - - Lasioglossum rohweri* Ellis, 1915 ***) | U.S.A | [30] |
| Ants (*Crematogaster* **)  Bees (Honey bees; *Apis mellifera ****, bumblebees; *Bombus* ***, leafcutting bees; Megachilidae *, halictid bees; *Augochlora ***, *Augochloropsis ***)  Birds (Hummingbirds; Ruby-throated*; Archilochus colubris ****)  Beetles (Marsh weevils; white dodder weevil *Smicronyx quadrifer* Blatchley, 1916 #, soldier beetles; Cantharidae #, leaf beetles; *Rhabdopterus praetexus* #, spotted cucumber beetle; *Diabrotica undecimpuctata* #, northern corn roothworm; *D. longicornis* #)  Flies (Green blow flies; *Lucilia* #, *Phaenicia* #, miners flies; *Agromyza borealis* Malloch, 1913 % [produce linear blotch mines], sawflies; *Aglaostigma semiluteum* Norton, 1862 [larvae] $, gall midges; + *Lasioptera fulva* [larvae] %, *Mycodiplosis impatientis* Felt, 1908 [larvae] % [*Lasioptera* and *Mycodiplosis* produce swollen stem galls])  Gamebirds (Ruffed grouse; *Bonasa umbellus* Linnaeus, 1776 &, ring-necked pheasants; *Phasianus colchicus* [Linnaeus](http://es.wikipedia.org/wiki/Carlos_Linneo)‎, 1758‎ &, northern bobwhites *Colinus virginianus* [Linnaeus](http://es.wikipedia.org/wiki/Carlos_Linneo)‎, 1758 &‎ [relish the seeds])  Grasshoppers #  Katydids #  Mices (White footed mice; *Peromyscus leucopus* Rafinesque, 1818 & [cache the seeds in their nests])  Mosquitoes (Gall midges**;** / *Schizomyia impatientis* %, [gall gnat larvae]; *Lasioptera impatientifolia* Felt, 1907 % [gall gnat larvae] swellings at leaf base or midrib folds)  Moths (Virginia tiger moth; *Spilosoma virginica* Fabricius, 1798 [caterpillars] #, whitestriped black mooth; *Trichodezia albovittata* Guenée, 1857 [caterpillars] #, tortricid moth; *Olethreutes agilana* Clemens, 1860 [caterpillars] $)  Stink bugs (Green stink bug; !*Chinavia hilaris* Say, 1832 [nymphs] #)  Shrews (Short tailed shrew; *Blarina brevicauda* Say, 1823 & [they cache the seeds in their nests])  Syrphid flies (*Rhingia nasica* Say, 1823 **, *Platycheirus ***)  Wasps (Eastern yellowjacket; *Vespula maculifrons* ***) | U.S.A | [31] |
| Birds (Hummingbirds *)  Bees (Bumblebees; Half-black bumblebee; *B. vagans* ***) | U.S.A | [32] |
| Birds (Hummingbirds; Ruby-throated; *Archilochus colubris ****)  Bees (Honey bees; *Apis mellifera* *, bumblebees; *Bombus* spp*.* *)  Wasps (*Vespula* spp*.* *) | U.S.A | [33] |
| Bees (Bumblebees; *Bombus* sp*. *,* honey bees; *Apis mellifera *,* sweat bees; *Augochlorella* spp*.**, *Augochlora* spp*.**) | U.S.A | [34] |
| Bees (Honey bees *****) | U.S.A | [35] |
| Birds (Hummingbirds; Ruby-throated spp. *, *Archilochus colubris* *)  Bees (Bumblebees; *Bombus vagans* ***) | U.S.A | [36] |
| Birds (Hummingbirds; Ruby-throated; *Archilochus colubris ****)  Bees (Bumblebees *) | U.S.A | [37] |
| Aphids #  Bees (bumble bees *, small bees *)  Beetles (Chrysomelid beetles #)  Deers (White-tailed deer; *Odocoileus virginianus* Zimmermann, 1780 #)  Grasshoppers #  Leaf miners # | U.S.A | [38] |
| Aphids #  Bees (Bumblebees; *Bombus* spp. *, honey bees; *Apis mellifera* *, sweat bees; ° *Lasioglossum rohweri* *)  Beetles (Chrysomelid beetles #)  Birds (Hummingbirds; Ruby-throated*; Archilochus colubris ****)  Deers (White-tailed deer; *Odocoileus virginianus* #)  Grasshoppers #  Katydids #  Leaf miners # | U.S.A | [39] |
| Bees (Bumblebees; Half-black bumblebee; *Bombus vagans* *, honey bees; *Apis mellifera* *, sweat bees; *Augochlorella striata* *) | U.S.A | [40] |
| Bees (Honey bees; *Apis mellifera* ***) | U.S.A | [41] |
| Butterflies (Monarch; *Danaus plexippus* Linnaeus, 1758 **, Pearl Cresent; *Phyciodes tharos* Drury, 1773 **, Pipevine Swallowtail; *Battus philenor* Linnaeus, 1771 **, Spicebush Swallowtail; *Papilio troilus* Linnaeus, 1758 **) | U.S.A | [42] |
| Bees (Bumblebees; *Bombus impatiens ****, *Bombus* spp. ***, honey bees; *Apis mellifera ****)  Birds (Hummingbirds *)  Wasps * | U.S.A | [43] |
| Bees (Bumble bees; Common eastern bumble bee; *Bombus impatiens* *, Half-black bumblebee; *B. vagans **, Yellow bumblebee; *B. fervidus* *), honey bees (*Apis mellifera* *)  Birds (Ruby-throated hummingbirds; *Archilochus colubris* *)  Wasps (Eastern yellowjacket; *Vespula maculifrons* *)  Bees ** and wasps ** | U.S.A | [44] |
| Ants (Formicidae; *Formica* spp. **)  Bees (Bumblebees; *Bombus* spp. *, honey bees; *Apis mellifera **, sweet bees; *Augochlora* spp. **)  Beetles (Japanese beetle; *Popillia japonica #*)  Mosquitoes (Gall midges; *Cecidomyia fulva* %, *Schizomyia impatientis* %)  Other generalist herbivores also eat flowers # | U.S.A | [45] |
| Beetles (Japanese beetle; *Popillia japonica #*)  Mosquitoes (Gall midges; Jewelweed midge; *Schizomyia impatientis* %)  Other generalist herbivores eat flowers # | U.S.A | [46] |
| Ants **  Bees (Bumble bees; *Bombus* sp. *, halictid bees **, honey bees; *Apis mellifera* *)  Beetles (Japanese beetle; *Popillia japonica* # [facultative florivore])  Spiders (Goldenrod Crab Spider; *Misumena vatia* Clerck, 1757 ⧫)  Wasps (Eastern yellowjacket; *Vespula maculifrons* **)  Other generalist herbivores # | U.S.A | [47] |
| Bees (Bumble bees; Common eastern bumble bee; *Bombus impatiens* *, another bee species *) | Canada | [48] |
| Honey bees *** | Canada | [49] |
| Bees * | U.S.A | [50] |

* Pollinators. ** Robber/thieves species (primary or secondary) (including the action of consuming only nectar in whatever part of saccate sepal). *** Pollinators and robbers/thieves species (primary or secondary) (including the action of consuming nectar in whatever part of saccate sepal). # Plant consumer species (including any part of the plant). % Parasitism: Flower/leaf gall species and linear blotch mines species. ⧫ Pollinators predators inhabiting plant organisms. & Other activities. $ No specific information. - Referred by Robertson (1928) as “*Bombus americanorum”. - -* Referred by Robertson (1928) as “*Chloralictus versatus*”. - - - Referred by Robertson (1928) as “*Oxystoglossa confusa”. - - - -* Referred by Schemske (1978) as “*Dactynotus impatiensicolens”*. - - - - - Referred by Schemske (1978) as “*Lasioptera impatientis*”. - - - - - - Referred by Schemske (1978) as “*Cecidomyia impatientis”.* - - - - - - - Referred by Schemske (1978) as “*Acrosternum hilare”. - - - - - - - -* Referred by Wilson & Thompson (1991) as “*Dialictus rohweri* “. / Referred by Eastman (1995) as “*Cecidomyia impatientis”*.! Referred by Eastman (1995) as “*Acrosternum hilare*”*. °* Referred by Steets (2005) as “*Dialictus rohweri* “. + Taxonomic authority unknown. ~Record of hummingbird, probably in *I. capensis* (according to the color description and mottling visualized in the picture), in an image titled: “*A Branch of the Humming-Bird Tree*.”

**References**

1. Josselyn J, Widdowes G. New-Englands rarities discovered : in birds, beasts, fishes, serpents, and plants of that country : together with the physical and chyrurgical remedies wherewith the natives constantly use to cure their distempers, wounds, and sores : also a perfect description of an Indian squa, in all her bravery : with a poem not improperly conferr'd upon her : lastly, a chronological table of the most remarkable passages in that country amongst the English : illustrated with cuts. London: Printed for G. Widdowes at the Green Dragon in St. Paul's Church-yard; 1672.

2. Bailey WW. Pine Barren Plants in Rhode Island. Bull Torrey Bot Club. 1880; 7(9): 98-99.

3. Beal W. Fertilization of flowers by hummingbirds. Am Nat. 1880; 14: 126-127.

4. Trelease W. Impatiens fulva, Action of Bees Toward. Bull Torrey Bot Club. 1880; 7(2): 20-21.

5. Lounsberry A. Southern Wild Flowers and Trees: Together with Shrubs, Vines and Various Forms of Growth Found Through the Mountains, the Middle District and the Low Country of the South: FA Stokes Company; 1901.

6. Weatherby CA. Color forms of Impatiens Biflora. Rhodora. 1917; 19(223): 115-118.

7. Carrol F. The development of the chasmogamous and cleistogamous flowers of Impatiens fulva (Balsaminaceae). Contributions from the University of Pennsylvania Botanical Laboratory. 1919; 4: 144-183.

8. Frost SW. A study of the leaf-mining Diptera of North America: Cornell University; 1924.

9. Robertson C. Flowers and insects; lists of visitors of four hundred and fifty-three flowers. Lancaster: The Scientific Press Printing Co; 1928.

10. Saunders AA. Ecology of the birds of Quaker Run Valley, Allegany State Park, New York: University of the state of New York; 1936.

11. James RL. Some Hummingbird Flowers East of the Mississippi. Castanea. 1948; 13(3): 97-109.

12. Martin A, Zim H, Nelson A. American Wildlife and Plants Dover Publications. Inc New York, NY ix. 1951; 500.

13. Hummel CM. The Biology, Ecology, and Description of the Jewelweed Flower-bud Midge Schizomyia Impatientis (Osten Sacken): Cornell University; 1956.

14. Pojar J. Hummingbird flowers of British Columbia. Syesis. 1975; 8: 25-27.

15. Wood CE. The Balsaminaceae in the southeastern United States. J Arnold Arbor. 1975; 56(4): 413-426.

16. Heinrich B. The Foraging Specializations of Individual Bumblebees. Ecol Monogr. 1976; 46(2): 105-128. https://doi.org/10.2307/1942246.

17. Heinrich B. Resource Partitioning Among Some Eusocial Insects: Bumblebees. Ecology. 1976; 57(5): 874-889. https://doi.org/10.2307/1941054.

18. Russell AE. Biosystematics of the genus Impatiens in northeastern North America. 1976.

19. Rust RW. Pollination in Impatiens capensis and Impatiens pallida (Balsaminaceae). Bull Torrey Bot Club. 1977; 104(4): 361-367. https://doi.org/10.2307/2484781.

20. Schemske DW. Evolution of Reproductive Characteristics in Impatiens (Balsaminaceae): The Significance of Cleistogamy and Chasmogamy. Ecology. 1978; 59(3): 596-613. https://doi.org/10.2307/1936588.

21. Buening SM. Biosystematic Studies of Impatiens pallida and Impatiens biflora (Balsaminaceae) 1979.

22. Leck MA. Germination behavior of Impatiens capensis Meerb.(Balsaminaceae). Bartonia. 1979; (46): 1-14.

23. Rust RW. Pollination of Impatiens capensis: Pollinators and Nectar Robbers. J Kans Entomol Soc. 1979; 52(2): 297-308.

24. Bertin RI. The Ruby-throated Hummingbird and its major food plants: ranges, flowering phenology, and migration. Can J Zool. 1982; 60(2): 210-219. https://doi.org/10.1139/z82-029.

25. Bell G, Lefebvre L, Giraldeau LA, Weary D. Partial preference of insects for the male flowers of an annual herb. Oecologia. 1984; 64(3): 287-294. https://doi.org/10.1007/BF00379123.

26. Laverty TM, Plowright RC. Competition between Hummingbirds and Bumble Bees for Nectar in Flowers of Impatiens biflora. Oecologia. 1985; 66(1): 25-32.

27. Zimmerman M, Cook S. Pollinator foraging, experimental nectar-robbing and plant fitness in Impatiens capensis. Am Midl Nat. 1985: 84-91.

28. Randall Jr JL. Pollination ecology of the simultaneously flowering Impatiens capensis and I. pallida: Virginia Polytechnic Institute and State University; 1988.

29. Dubé D. Flight path of pollinators foraging on impatiens: decision rules and their implications for gene flow. Montréal, Québec, Canada: McGill University; 1989.

30. Wilson P, Thomson JD. Heterogeneity Among Floral Visitors Leads to Discordance Between Removal and Deposition of Pollen. Ecology. 1991; 72(4): 1503-1507. https://doi.org/10.2307/1941124.

31. Eastman JA. The book of swamp and bog: trees, shrubs, and wildflowers of the eastern freshwater wetlands: Stackpole Books; 1995.

32. Wilson P. Selection for pollination success and the mechanical fit of Impatiens flowers around bumblebee bodies. Biol J Linn Soc. 1995; 55(4): 354-383. https://doi.org/10.1016/S0024-4066(05)80003-3.

33. Hurlbert AH, Hosoi SA, Temeles EJ, Ewald PW. Mobility of Impatiens capensis flowers: effect on pollen deposition and hummingbird foraging. Oecologia. 1996; 105(2): 243-246. https://doi.org/10.1007/BF00328553.

34. Walters BB, Stiles EW. Effect of Canopy Gaps and Flower Patch Size on Pollinator Visitation of Impatiens capensis. Bull Torrey Bot Club. 1996; 123(3): 184-188. https://doi.org/10.2307/2996793.

35. Grabas GP, Laverty TM. The effect of purple loosestrife (Lythrum salicaria L.; Lythraceae) on the pollination and reproductive success of sympatric co-flowering wetland plants. Ecoscience. 1999; 6(2): 230-242.

36. Pan IL. Pollinator-mediated selection on a floral trait: spur variation in Impatiens capensis: Amherst College; 1999.

37. Travers SE, Temeles EJ, Pan I. The relationship between nectar spur curvature in jewelweed (Impatiens capensis) and pollen removal by hummingbird pollinators. Canad J Bot. 2003; 81(2): 164-170. https://doi.org/10.1139/b03-014.

38. Steets JA, Ashman T-L. Herbivory Alters the Expression of a Mixed-Mating System. Am J Bot. 2004; 91(7): 1046-1051.

39. Steets JA. Antagonists and mixed mating: consequences for the demography of Impatiens capensis (Balsaminaceae): University of Pittsburgh; 2005.

40. Steets JA, Hamrick JL, Ashman T-L. Consequences of vegetative herbivory for maintenance of intermediate outcrossing in an annual plant. Ecology. 2006; 87(11): 2717-2727. https://doi.org/10.1890/0012-9658(2006)87[2717:COVHFM]2.0.CO;2.

41. Zika PF. The Status of Impatiens capensis (Balsaminaceae) on the Pacific Northwest Coast. J Torrey Bot Soc. 2006; 133(4): 593-600.

42. Foote K. Orange Jewelweed (Impatiens capensis Meerburgh). New York Flora Association Newsletter. 2007; 18: 10-18.

43. Young HJ, Dunning DW, von Hasseln KW. Foraging Behavior Affects Pollen Removal and Deposition in Impatiens Capensis (Balsaminaceae). Am J Bot. 2007; 94(7): 1267-71.

44. Young HJ. Selection on Spur Shape in Impatiens capensis. Oecologia. 2008;156(3):535-43.

45. Gorden NLS. Interactions between floral mutualists and antagonists, and consequences for plant reproduction: University of Massachusetts Amherst; 2013.

46. Gorden NLS, Adler LS. Abiotic conditions affect floral antagonists and mutualists of Impatiens capensis (Balsaminaceae). Am J Bot. 2013; 100(4): 679-689.

47. Gorden NLS, Adler LS. Florivory shapes both leaf and floral interactions. Ecosphere. 2016; 7(6): e01326. https://doi.org/10.1002/ecs2.1326.

48. McDonald S, Caruso CM. Simulated Nectar Robbing Does Not Affect Pollinator-Mediated Selection on Floral Traits of Impatiens capensis. Int J Plant Sci. 2019; 180(8): 922-927. https://doi.org/10.1086/704830.

49. Barker CA, Sargent RD. Pollination Services to Impatiens capensis (Balsaminaceae) Are Maintained across an Urbanization Gradient. Int J Plant Sci. 2020; 181(9): 937-944. https://doi.org/10.1086/710488.

50. Barfknecht DF, Gibson DJ. Are metapopulation species drivers of metacommunity structure in sandstone outcrop communities? J Veg Sci. 2023; 34(1): e13167. <https://doi.org/10.1111/jvs.13167>.
