# Supplementary material for: Pollination biology of Impatiens capensis Meerb. in non-native range
Source: PLoS One. 2024 Jun 20;19(6):e0302283. doi: 10.1371/journal.pone.0302283 (PMC11189253; doi:10.1371/journal.pone.0302283)
Supplement: S2 Table — (DOCX) [file pone.0302283.s003.docx]

S2 Table. Pollinators and nectar robbers of *Impatiens capensis* noticed in Poland*.*

| **BIN** | **N** | **n** | **Species ID** | **site** | | | | **MP** | **status** | **Fi (%)** |
| --- | --- | --- | --- | --- | --- | --- | --- | --- | --- | --- |
|  |  |  |  | Po | Sz | Sw | Bu |  |  |  |
| ACZ9928 | 1 | 1 | Coleoptera: Elateridae: *Synaptus filiformis* |  | 1 |  |  | C | P | 25 |
| ABA6492 | 1 | 1 | Diptera: Anthomyiidae: *Hylemya vagans* |  |  | 1 |  | S | P | 25 |
| ACD5684 | 1 | 1 | Diptera: Sciomyzidae: *Tetanocera silvatica* |  | 1 |  |  | S | P | 25 |
| AAY9803 | 1 | 1 | Diptera: Syrphidae: *Tropidia scita* | 1 |  |  |  | S | P | 25 |
| AAC6833 | 9 | 8 | Diptera: Syrphidae: *Episyrphus balteatus* |  | 9 |  |  | S | P | 25 |
| AEJ8030 | 1 | 1 | Diptera: Syrphidae: *Helophilus trivittatus* |  |  | 1 |  | S | P | 25 |
| AAA7374 | 1 | 1 | Diptera: Syrphidae: *Sphaerophoria scripta* |  |  |  | 1 | S | P | 25 |
| ABY7543 | 1 | 1 | Hemiptera: Miridae: *Adelphocoris quadripunctatus* |  |  |  | 1 | PS | T | 25 |
| AAA2326 | 4 | 3 | Hymenoptera: Apidae: *Apis mellifera* |  | 1 | 3 |  | CS | P | 50 |
| AAD2566 | 1 | 1 | Hymenoptera: Apidae: *Bombus hortorum* |  |  | 1 |  | CS | P | 25 |
| AAC4378 | 22 | 17 | Hymenoptera: Apidae: *Bombus pascuorum* | 1 | 12 | 7 | 2 | CS | P | 100 |
| AAB1062 | 1 | 1 | Hymenoptera: Apidae: *Bombus terrestris* |  |  | 1 |  | CS | P | 25 |
| AAD4214 | 1 | 1 | Hymenoptera: Ichneumonidae: *Diplazon laetatorius* |  | 1 |  |  | C | P | 25 |
| n/a | 1 | 0 | Hymenoptera: Tenthredinidae: *Athalia rosae* | 1 |  |  |  | C | P | 25 |
| AAM3988 | 5 | 5 | Hymenoptera: Vespidae: *Dolichovespula saxonica* | 2 | 1 | 2 |  | C | P | 75 |
| AAG9055 | 2 | 2 | Hymenoptera: Vespidae: *Vespula germanica* |  |  | 2 |  | C | P | 25 |
| AAM2781 | 12 | 12 | Hymenoptera: Vespidae: *Vespula vulgaris* | 1 | 7 | 4 |  | C | P | 75 |
| ACF8169 | 1 | 1 | Mecoptera: Panorpidae: *Panorpa vulgaris* |  | 1 |  |  | C | P | 25 |
| n/a | 2 | 0 | Orthoptera: Tettigoniidae: *Leptophyes punctatissima* |  | 2 |  |  | C | T | 25 |
| AAN9260 | 1 | 1 | Stylommatophora: Succineidae: *Succinea putris* |  | 1 |  |  | CS | T | 25 |

BIN – Barcode Index Number, n/a – barcode not assigned, only morphological identification; N – total number of individuals collected; n – number of obtained sequences; Species ID – taxonomical assignment of the species; site – population acronyms: Po – Police, Sz – Szczecin-Zdroje, Sw – Święta, B – Budzień; MP – mouthpart: C, chewing; S, sponging; PS, piercing and sucking; CS, chewing and sucking; P – pollinator; T – pollen/nectar robber;
