# Supplementary material for: Pollination biology of Impatiens capensis Meerb. in non-native range
Source: PLoS One. 2024 Jun 20;19(6):e0302283. doi: 10.1371/journal.pone.0302283 (PMC11189253; doi:10.1371/journal.pone.0302283)
Supplement: S3 Table — (DOCX) [file pone.0302283.s004.docx]

S3 Table. Fuzzy coded matrix

| **Order** | **Family** | **Species** | **Data** | **code** | **ST.P** | **ST.T** | **MP.B** | **MP.C** | **MP.S** | **MP.L** | **MP.CS** | **MP.PS** | **SI.1** | **SI.2** | **SI.3** | **SI.4** |
| --- | --- | --- | --- | --- | --- | --- | --- | --- | --- | --- | --- | --- | --- | --- | --- | --- |
| Hymenoptera | Apidae | Apis mellifera | Both | amell | 3 | 0 | 0 | 0 | 0 | 0 | 3 | 0 | 0 | 2 | 2 | 0 |
| Hymenoptera | Halictidae | Augochloropsis metallica | Literature | ameta | 2 | 2 | 0 | 0 | 0 | 0 | 3 | 0 | 0 | 2 | 2 | 0 |
| Hymenoptera | Halictidae | Augochlora pura | Literature | apura | 2 | 2 | 0 | 0 | 0 | 0 | 3 | 0 | 0 | 3 | 0 | 0 |
| Hemiptera | Miridae | Adelphocoris quadripunctatus | This | aquad | 0 | 3 | 0 | 0 | 0 | 0 | 0 | 3 | 0 | 2 | 1 | 0 |
| Hymenoptera | Tenthredinidae | Athalia rosae | This | arosa | 3 | 0 | 0 | 3 | 0 | 0 | 0 | 0 | 1 | 2 | 0 | 0 |
| Hymenoptera | Halictidae | Augochlorella striata | Literature | astri | 3 | 0 | 0 | 0 | 0 | 0 | 3 | 0 | 2 | 2 | 0 | 0 |
| Hymenoptera | Apidae | Bombus affinis | Literature | baffi | 2 | 2 | 0 | 0 | 0 | 0 | 3 | 0 | 0 | 1 | 2 | 2 |
| Hymenoptera | Apidae | Bombus fervidus | Literature | bferv | 2 | 2 | 0 | 0 | 0 | 0 | 3 | 0 | 0 | 0 | 2 | 2 |
| Hymenoptera | Apidae | Bombus griseocollis | Literature | bgris | 2 | 2 | 0 | 0 | 0 | 0 | 3 | 0 | 0 | 0 | 3 | 0 |
| Hymenoptera | Apidae | Bombus hortorum | This | bhort | 3 | 0 | 0 | 0 | 0 | 0 | 3 | 0 | 0 | 1 | 2 | 2 |
| Hymenoptera | Apidae | Bombus impatiens | Literature | bimpa | 2 | 2 | 0 | 0 | 0 | 0 | 3 | 0 | 0 | 2 | 2 | 1 |
| Hymenoptera | Apidae | Bombus pascuorum | This | bpasc | 3 | 0 | 0 | 0 | 0 | 0 | 3 | 0 | 0 | 2 | 2 | 0 |
| Hymenoptera | Apidae | Bombus pensylvanicus | Literature | bpens | 3 | 0 | 0 | 0 | 0 | 0 | 3 | 0 | 0 | 0 | 2 | 2 |
| Hymenoptera | Apidae | Bombus perplexus | Literature | bperp | 0 | 3 | 0 | 0 | 0 | 0 | 3 | 0 | 0 | 0 | 3 | 0 |
| Hymenoptera | Apidae | Bombus ternarius | Literature | btern | 2 | 2 | 0 | 0 | 0 | 0 | 3 | 0 | 0 | 2 | 2 | 0 |
| Hymenoptera | Apidae | Bombus terrestris | This | bterre | 3 | 0 | 0 | 0 | 0 | 0 | 3 | 0 | 0 | 0 | 2 | 2 |
| Hymenoptera | Apidae | Bombus terricola | Literature | bterri | 0 | 3 | 0 | 0 | 0 | 0 | 3 | 0 | 0 | 2 | 2 | 0 |
| Hymenoptera | Apidae | Bombus vagans | Literature | bvaga | 2 | 2 | 0 | 0 | 0 | 0 | 3 | 0 | 0 | 0 | 3 | 0 |
| Hymenoptera | Formicidae | Camponotus sp | Literature | casp | 0 | 3 | 3 | 0 | 0 | 0 | 0 | 0 | 1 | 2 | 2 | 0 |
| Hymenoptera | Formicidae | Crematogaster sp. | Literature | crsp | 2 | 2 | 3 | 0 | 0 | 0 | 0 | 0 | 3 | 0 | 0 | 0 |
| Hymenoptera | Ichneumonidae | Diplazon laetatorius | This | dlaet | 3 | 0 | 0 | 3 | 0 | 0 | 0 | 0 | 3 | 0 | 0 | 0 |
| Coleoptera | Chrysomelidae | Diabrotica longicornis | Literature | dlong | 2 | 2 | 3 | 0 | 0 | 0 | 3 | 0 | 3 | 0 | 0 | 0 |
| Hymenoptera | Vespidae | Dolichovespula saxonica | This | dsaxo | 3 | 0 | 0 | 3 | 0 | 0 | 0 | 0 | 0 | 0 | 3 | 0 |
| Coleoptera | Chrysomelidae | Diabrotica undecimpunctata | Literature | dunde | 2 | 2 | 3 | 0 | 0 | 0 | 0 | 0 | 3 | 0 | 0 | 0 |
| Diptera | Syrphidae | Episyrphus balteatus | This | ebalt | 3 | 0 | 0 | 0 | 3 | 0 | 0 | 0 | 0 | 2 | 2 | 0 |
| Hymenoptera | Formicidae | Formica spp | Literature | fospp | 2 | 2 | 3 | 0 | 0 | 0 | 0 | 0 | 0 | 3 | 0 | 0 |
| Hymenoptera | Halictidae | Halictus sp. | Literature | hasp | 0 | 3 | 0 | 0 | 0 | 0 | 3 | 0 | 0 | 0 | 3 | 0 |
| Diptera | Syrphidae | Helophilus trivittatus | This | htriv | 3 | 0 | 0 | 0 | 3 | 0 | 0 | 0 | 0 | 0 | 2 | 2 |
| Diptera | Anthomyiidae | Hylemya vagans | This | hvaga | 3 | 0 | 0 | 0 | 3 | 0 | 0 | 0 | 2 | 2 | 0 | 0 |
| Orthoptera | Tettigoniidae | Leptophyes punctatissima | This | lpunc | 0 | 3 | 0 | 3 | 0 | 0 | 0 | 0 | 0 | 1 | 2 | 2 |
| Hymenoptera | Halictidae | Lasioglossum rohweri | Literature | lrohw | 3 | 0 | 0 | 0 | 0 | 0 | 3 | 0 | 3 | 0 | 0 | 0 |
| Diptera | Syrphidae | Platycheirus sp. | Literature | plsp | 2 | 2 | 0 | 0 | 0 | 3 | 0 | 0 | 0 | 3 | 0 | 0 |
| Mecoptera | Panorpidae | Panorpa vulgaris | This | pvulg | 3 | 0 | 0 | 3 | 0 | 0 | 0 | 0 | 0 | 0 | 3 | 0 |
| Diptera | Syrphidae | Rhingia nasica | Literature | rnasi | 2 | 2 | 0 | 0 | 0 | 3 | 0 | 0 | 2 | 2 | 0 | 0 |
| Coleoptera | Elateridae | Synaptus filiformis | This | sfili | 3 | 0 | 0 | 3 | 0 | 0 | 0 | 0 | 0 | 2 | 2 | 0 |
| Stylommatophora | Succineidae | Succinea putris | This | sputr | 0 | 3 | 0 | 0 | 0 | 0 | 3 | 0 | 0 | 0 | 0 | 3 |
| Diptera | Syrphidae | Sphaerophoria scripta | This | sscri | 3 | 0 | 0 | 0 | 3 | 0 | 0 | 0 | 1 | 2 | 2 | 0 |
| Diptera | Syrphidae | Tropidia scita | This | tscit | 3 | 0 | 0 | 0 | 3 | 0 | 0 | 0 | 1 | 2 | 2 | 0 |
| Diptera | Sciomyzidae | Tetanocera silvatica | This | tsilv | 3 | 0 | 0 | 0 | 3 | 0 | 0 | 0 | 2 | 2 | 0 | 0 |
| Hymenoptera | Vespidae | Vespula acutifrons | Literature | vacut | 0 | 3 | 0 | 3 | 0 | 0 | 0 | 0 | 3 | 0 | 0 | 0 |
| Hymenoptera | Vespidae | Vespula germanica | This | vgerm | 3 | 0 | 0 | 3 | 0 | 0 | 0 | 0 | 0 | 0 | 2 | 2 |
| Hymenoptera | Vespidae | Vespula maculifrons | Literature | vmacu | 0 | 3 | 0 | 3 | 0 | 0 | 0 | 0 | 0 | 0 | 2 | 2 |
| Hymenoptera | Vespidae | Vespula vulgaris | This | vvulg | 3 | 0 | 0 | 3 | 0 | 0 | 0 | 0 | 0 | 0 | 3 | 0 |
